# A cross-sectional study on the comparison of serum SIRT-1 and MMP-9 levels of patients with bronchiectasis and healthy controls

**DOI:** 10.12669/pjms.41.4.10877

**Published:** 2025-04

**Authors:** Deniz Bakir, Mustafa D. Bedir, Dilara Ulger Ozbek, Zehra Seyfikli

**Affiliations:** 1 Deniz Bakir Medical Faculty Hospital, Biochemistry Laboratory, Sivas Cumhuriyet University, Sivas, Turkey; 2 Mustafa D. Bedir Yıldızeli Vocational School, Chemistry and Chemical Processing Technologies, Medical Biochemistry, Sivas Cumhuriyet University, Sivas, Turkey; 3 Dilara Ulger Ozbek Sivas Cumhuriyet University, Sivas, Turkey; 4 Zehra Seyfikli Medicine Faculty, Department of Chest Diseases, Yozgat Bozok University, Yozgat, Turkey

**Keywords:** Bronchiectasis, ELISA, MMP-9, SIRT-1

## Abstract

**Background & Objectives:**

Bronchiectasis is the permanent enlargement of the bronchi following damage to the respiratory tract (bronchi) in the lungs. Bronchiectasis not associated with cystic fibrosis is gaining an increasing place among chronic respiratory diseases worldwide. The purpose of this study was to identify the levels of MMP-9, known to cause bronchial damage in chronic pulmonary illness, and SIRT-1, an anti-aging and anti-infective regulatory protein, in patients with bronchiectasis and to evaluate the importance of these biomarkers in diagnosis.

**Methods:**

This cross-sectional study was conducted in the Chest Diseases Clinic of Sivas Cumhuriyet University Medical Faculty Hospital between November 2020 and September 2022. We recruited 30 patients with bronchiectasis whose diagnosis was verified by high-resolution chest CT scan and 30 healthy controls. SIRT-1 and MMP-9 levels in the serum of the study group were determined by the ELISA method.

**Results:**

SIRT-1 and MMP-9 concentrations were found to be statistically significant. In comparison to the control group, it was observed that the bronchiectasis group had a lower serum SIRT-1 levels (p<0.001). The bronchiectasis group had higher serum MMP-9 values than the control group (p<0.001). Age-related differences in SIRT-1 and MMP-9 concentrations were not observed. No correlation was found between MMP-9 and SIRT-1. The results of Receiver Operating Characteristic (ROC) analysis indicated that MMP-9 has relatively high sensitivities.

**Conclusions:**

We concluded that, higher inflammation elevates MMP-9 levels while decreasing SIRT-1 levels. MMP-9 and SIRT-1 may be potential biomarkers in the diagnosis of bronchiectasis.

## INTRODUCTION

Bronchiectasis, is a complex, inflammatory, respiratory disease that is unrelated to cystic fibrosis. It is commonly regarded as an irreversible bronchial widening.^1^ It is distinguished by an endless loop of bronchiectasis, bacterial colonialism, recurring infections, persistent cough and sputum symptoms, and increased the disease’s severity.^2^ Whatever the underlying cause, bronchiectasis is caused by the interplay of host-impaired defenses and environmental variables and lung inflammation.^3^ Intense and recurrent inflammation in bronchiectasis may cause increased lung destruction by various mechanisms. In the airway lumen of bronchiectasis, neutrophilic inflammation is predominant. Neutrophils secrete lytic enzymes such as neutrophil elastase, myeloperoxidase, which are likely to have an impact in the recurrent airway injury of bronchiectasis.^4^ Many studies have found a link between the action of neutrophil elastase and proinflammatory cytokine levels that increase in patients with bronchiectasis and correlate with lung functions.^5^,^6^

Matrix mettaloproteinases (MMPs) are a group of nine or more highly homologous Zn endopeptidases that cleave the majority, if not all of the extracellular matrix elements.^7^ Due to their ability to degrade all extracellular matrix components, they are stably expressed in repaired tissues and are not usually found in healthy lung tissue.^8^ In recent times, it has been reported that changes in the balance of proteases and anti-elastases are effective in the pathogenesis of chronic lung diseases. MMPs, one of the neutrophil-derived proteases, can cause bronchial damage in chronic lung diseases including bronchiectasis.^4^ MMP-9 is the most popular protease among many MMPs because of its predominance in alveolar tissues and its easy detection and quantification. It has been suggested that they can be utilized as biomarkers in chronic respiratory diseases.^9^

Sirtuin protein family is a crucial gene silencing complex belonging to histone deacetylases (HDAC) class III and is found in all eukaryotes as an essential regulator of lifespan, anti-stress, anti-inflammation and anti-aging.^10^ Also, its principle differs from that of other HDACs, because it necessitates the cofactor nicotinamide adenine dinucleotide (NAD^+^) to do deacetylation; this deactivates ε-acetylamino by cutting out the acetyl-lysines of histones, thence gene transcription is inhibited.^11^ The first discovered sirtuin family member was SIRT-1, mainly found in the nucleus and cytoplasm.^12^ SIRT-1, as an HDAC protein, regulates stress resistance, DNA repair, differentiation, inflammation, oxidative stress, metabolism, apoptosis, and senescence.^13^ Studies have reported a role for SIRT- in many lung diseases.^14^,^15^

This current study in bronchiectasis aimed to evaluate the role of SIRT-1, which may have a critical function in regulating inflammation and other pathophysiological pathways, and the neutrophil-derived protease MMP-9, which is known to cause bronchial damage in various lung diseases.

## METHODS

In this cross-sectional study, we screened 110 participants who were excluded due to additional upper respiratory tract disease between November 2020 and September 2022.

### Ethical Approval:

The study protocol was approved by Sivas Cumhuriyet University Clinical Research Ethics Committee (Ref# 2020-02/01, Date: February 25, 2020). In addition, all participants provided written informed consent.

The clinical trials registration number of the study is NCT06589492. Finally, we included 30 patients with bronchiectasis and 30 controls. In the overall study population of 60 subjects, who applied to the Sivas Cumhuriyet University Faculty of Medicine Research and Practice Hospital Chest Diseases Clinic. The study population was selected regardless of age and gender. We selected bronchiectasis patients who had symptoms (cough, sputum production) and had their diagnosis confirmed by a chest high-resolution CT scan within the previous year. We excluded individuals with bronchiectasis associated with cystic fibrosis and who had a worsening episode (according to the *2017 European Respiratory Society expert consensus criteria*)^16^ or had taken antibiotics during the previous four weeks. We enrolled healthy controls through advertisements who had acceptable chest X-ray and spirometry results and no lower airway symptoms or serious systemic disorders. We eliminated individuals who were pregnant or nursing, as well as those with low comprehension.

### Samples collection and measurement of biochemical parameters:

Peripheral venous blood samples required for the study were collected in the morning after at least eight hours of night fasting. Five mL of whole blood was transferred in biochemistry gel tubes (VACUTTE, USA) and centrifuged for 15 minutes at 4000 rpm, after which the serums were separated into microcentrifuge tubes and labeled. The samples were kept at -80 °C until the working day.

According to the manufacturer’s instructions, serum SIRT-1 and MMP-levels were measured by a commercial ELISA kit (Elabscience Biotechnology Co., Ltd, China). The minimum detectable dose of human SIRT-1 ELISA kit (Catalog No: E-EL-H1546) is 0.19 ng/mL, whereas that of MMP-9 (Catalog No: E-EL-H6075) is 0.1 ng/mL. The detection range of ELISA kits for serum SIRT-1 and MMP-9 is 0.31-20 ng/mL and 0.16-10 ng/mL respectively.

### Statistical Analysis:

Statistical analyses were performed using Graph Pad Prism 6.01 (GraphPad Software Inc, USA) and Jamovi 2.3.28. The Kolmogorov-Smirnov test was used to distribute the data. According to the data distribution, if the data has a normal distribution, a parametric independent t test was used for analysis, and the results were given as the mean ± standard error of the mean (SEM). The non-parametric Mann-Whitney U test was employed for analysis in cases when the data did not conform to the normal distribution. The results were given as median (quartile 25–quartile 75%). The chi-squared tests was used for the categorical variables. For the correlation analysis, the Spearman analysis was used. Receiver operating characteristic (ROC) analysis was performed to evaluate the specificity and sensitivity of using serum MMP-9 and SIRT-1 as markers for bronchiectasis. Area under the curve (AUC), cut off value, PPV (positive predictive value), NPV (negative predictive value), sensitivity, specificity, and ROC curves visualized the data. Statistical significance was set at p < 0.05.

## RESULTS

This study examined bronchiectasis SIRT-1 and MMP-9 levels and its relationship to age and gender. The gender distribution of the study group is given below (Table-I). There was no discernible difference in MMP-9, SIRT-1, or age (Table-I).

**Table-I:** Age and gender distribution of study groups.

Variable	Groups			
		Bronchiectasis (n=30)	Control (n=30)	p
Gender	Male	18 (%60)	17 (%56.7)
	Female	12 (%40)	13 (%43.3)
Age		47.5 ± 9.6	48.3 ± 11.2	>0.005

Gender data were expressed as a percentage and age data were expressed as mean ± SD where appropriate.

Gender data were expressed as a percentage and age data were expressed as mean ± SD where appropriate.

**Fig.1:**
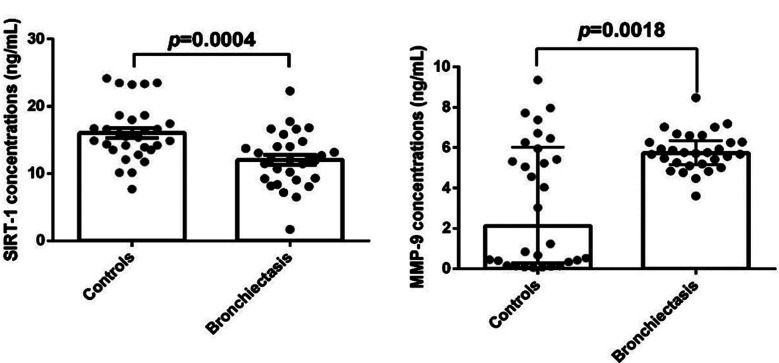
The statistically evaluated serum SIRT-1 and MMP-9 levels plots.

The focus of the study was to compare MMP-9 and SIRT-1 levels and the difference was found to be significant (Fig.1). The control group’s SIRT-1 levels were found to be significantly greater than those of the patient’s group (p<0.001). MMP-9 levels proved to be much greater and significant in the patient group as compared to the control group, in contrast to SIRT-1 (p=0.002) (Table-II).

**Table-II:** Comparison of SIRT-1 and MMP-9 levels between the groups.

Variable	Groups		p
	Bronchiectasis (n=30)	Control (n=30)	
SIRT-1 [ng/mL]	12.04 ± 0.74	16.05 ± 0.77	0.0004*
MMP-9 [ng/mL]	Median (Q1-Q3)	Median (Q1-Q3)	0.002*
	5.74 (5.17-6.35)	2.13 (0.30-6.03)	

* Data expressed as mean ± SEM, t test was used for differences between groups (p<0.005). Data were given as median (IQR1-IQR3), Q1: First quartile, Q3: Third quartile. Mann–Whitney U test for continuous variables were used to test group differences (*p*<0.005). (SIRT-1: Sirtuin-1, SEM: standard error of the mean, IQR: Interquartile range, MMP-9: Matrix metalloproteinase-9).

**Fig.2:**
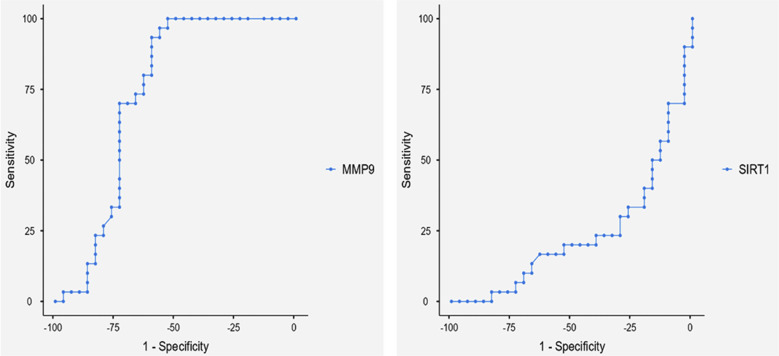
Evaluation of the diagnostic performance of SIRT-1 and MMP-9 variables predicting bronchiectasis patients.

Since the AUC value of SIRT-1 levels is less than <0.5 (AUC=0.238), its use as a marker in the diagnosis of bronchiectasis is not statistically significant. The ROC analysis of study showed a high sensitivity of MMP-9 for the diagnosis of bronchiectasis (Fig.2).

There is a negative (r= -0.192) but statistically insignificant (p=0.142) correlation found between MMP-9 and SIRT-1 levels. A statistically significant cut-off value for the diagnosis of bronchiectasis was obtained, according the ROC analysis of MMP-9 (Table-III).

**Table-III:** ROC analysis of MMP-9 in serum results that can predict bronchiectasis.

Risk factor	Cut off	p	%Sensitivity	% Specificity	%PPV	%NPV	Youden’s index	AUC
MMP-9	4.77	0.002	93.3	60	70	90	0.533	0.731

*Data are statistically significant (p < 0.05). The parameters are ranked according to their AUC values. (AUC: area under curve, PPV; positive predictive value, NPV: negative predicted value, MMP-9: Matrix metalloproteinase-9).

## DISCUSSION

This is the first study to examine serum MMP-9 and SIRT-1 levels together in individuals suffering from bronchiectasis. Serum SIRT-1 and MMP-9 levels were compared between healthy individuals and bronchiectasis patients, and the diagnostic sensitivity of these tests was assessed. The study found that serum MMP-9 levels were substantially higher in patients than in controls (p<0.05). SIRT-1 levels were considerably lower in bronchiectasis patients compared to the control group (p<0.05). Numerous studies have revealed that SIRT-1 and MMP-9 take part in a wide range of processes, both physiological and pathological, and that they have an inverse relationship. Researchers have shown that SIRT-1 levels decrease and MMP-9 levels increase, with a negative connection, in individuals with COPD^17^, ADHD^18^, and diabetic retinopathy.^19^ In line with this knowledge, our investigation found a negative connection between SIRT-1 and MMP-9, however it was not statistically significant.

Although the pathophysiology of bronchiectasis is not fully understood, neutrophil dysfunction in the airways is thought to be a critical component of the of this vicious loop of lung destruction. In airway inflammation, neutrophils are the most crucial cells. When exposed to an inflammatory stimulation, neutrophils induce serine proteases and matrix metalloproteases, which cause the extracellular media’s structure to be disrupted.^20^,^21^ Even in stable bronchiectasis airway secretions, prior investigations observed higher levels of many pro-inflammatory, neutrophil-derived cytokine.^22^ MMP’s are proteolytic molecules that are generated by neutrophils and alveolar macrophages as part of the inflammatory response in many pulmonary disorders including bronchiectasis. As a result, higher MMP-9 levels may represent airway damage and be linked with reduced pulmonary function.^4^ Only a few research studies have looked examined MMP-9 levels in bronchiectasis patients. Patients’ endobronchial samples with bronchiectasis revealed that neutrophil MMPs were overexpressed.^6^ In another investigation, MMP-9, as well as bacteria-derived collagenolytic proteases, were found in broncho alveolar lavage (BAL) samples from patients suffering from bronchiectasis.^23^

We found that MMP-9 levels were significantly increased in the serum of patients with bronchiectasis, and this result strengthens the findings of the reports cited here. These results might indicate a lung blockage and have anything to do with the localized pulmonary inflammatory condition. Taken together, these approaches lend evidence to MMP-9’s involvement as a crucial mediator in bronchiectasis. Additionally, it was observed that MMP-9 had high sensitivity (93%) in the serum of patients with bronchiectasis. We might speculate that serum MMP-9 concentrations may help in the diagnosis of bronchiectasis given the with high precision.

Given that inflammatory conditions are one of the most basic processes underlying the advancement of bronchiectasis, nuclear factor-kappa B (NFκB), a redox-sensitive transcriptional factor, has been found to increase the production of pro-inflammatory molecules. In a study conducted in COPD patients, it was observed that SIRT-1 activity and protein expression were decreased compared to healthy controls. Also, decreased SIRT-1 levels were associated with increased IL-8 and MMP-9 levels.^17^ We also found that serum levels of SIRT-1 significantly decreased in patients with bronchiectasis compared to the healthy controls, which was consistent with a previous study. In addition, there is an increase in MMP-9 levels in COPD^24^ and some inflammatory diseases.^25^ Inflammation results in reduced expression and activity of the NAD^+^-dependent deacetylase SIRT-1. As a result, even small variations in SIRT-1 expression and function can have a big influence on cellular responses.^26^

The results of the current study were consistent with previous research and comparable inflammatory lung disorders. Since it has been proven that SIRT-1 can affect inflammatory pathways by inhibiting NF-κB activity, it can be concluded that decreased SIRT-1 activity and high MMP-9 levels are associated with increased inflammatory conditions in bronchiectasis patients. Further research on the mechanisms controlling SIRT-1 expression and function is needed due to its apparent importance in the inflammatory response.

This work provides crucial information to illustrate the possible roles of these biomarkers in the pathophysiology of the illness by comparing the serum SIRT-1 and MMP-9 levels in bronchiectasis patients with those in healthy persons. The results might help us better understand how inflammatory and tissue-destructive processes relate to bronchiectasis, given the paucity of research on this topic in the literature. When analyzed from a clinical standpoint, it might offer a fresh viewpoint on whether SIRT-1 and MMP-9 can be employed as biomarkers in bronchiectasis patients. It might also serve as a crucial foundation for early disease diagnosis, prognosis tracking, and treatment approach customization.

This study’s primary merit lies in its contribution to the sparse literature by comparing blood SIRT-1 and MMP-9 levels between bronchiectasis patients and healthy controls. Furthermore, our study offers a thorough analysis to assess the connection between biomarkers and disease etiology and was carried out using properly chosen patient and control groups.

In certain areas, more research is required, even if this work is a valuable beginning point for comprehending the possible involvement of SIRT-1 and MMP-9 in the pathophysiology of bronchiectasis. To start, extensive, long-term follow-up research should be carried out to learn more about how these biomarkers affect the course and outcome of illness. Furthermore, research is required to assess the changes in SIRT-1 and MMP-9 levels in other bronchiectasis phenotypes, such as inflammatory or infectious forms. Lastly, prospective research is required to ascertain whether these biomarkers are appropriate for clinical application in terms of therapy response.

### Limitation:

It includes small sample size. Second, measuring many inflammatory indicators can help design more effective defenses. However, we did not need to assess inflammatory marker levels since our goal was to evaluate MMP-9 and SIRT-1 levels, examine their levels among the patient control group, and determine their relevance in the diagnosis of bronchiectasis. More in-depth research is intended to be done in light of these.

## CONCLUSION

Taking into account the levels in the control and patient groups, we concluded that elevated inflammation, which is anticipated to occur in bronchiectasis, may cause MMP-9 levels to increase and SIRT-1 levels to decrease. It was determined that MMP-9 might be reliable markers given their high sensitivity in the diagnosis of bronchiectasis, despite the fact that there was no discernible correlation between them.

### Author Contributions:

**DB, DUO, ZS,** and **MDB:** Developed the concept of the study.

**ZS, DB** and **DUO:** Were involved in data and sample collection.

**DB** and **DUO:** Were involved in measuring tests.

**DUO** and **MDB:** Were involved in data analysis. All authors reviewed the manuscript.

All authors have assumed full responsibility for the content of this work and have given their approval for its submission.
